# MANET: tracing evolution of protein architecture in metabolic networks

**DOI:** 10.1186/1471-2105-7-351

**Published:** 2006-07-19

**Authors:** Hee Shin Kim, Jay E Mittenthal, Gustavo Caetano-Anollés

**Affiliations:** 1 Department of Crop Sciences, University of Illinois at Urbana-Champaign, Urbana, IL 61801, USA; 2 Department of Cell and Developmental Biology, University of Illinois at Urbana-Champaign, Urbana, IL 61801, USA

## Abstract

**Background:**

Cellular metabolism can be characterized by networks of enzymatic reactions and transport processes capable of supporting cellular life. Our aim is to find evolutionary *patterns *and *processes *embedded in the architecture and function of modern metabolism, using information derived from structural genomics.

**Description:**

The Molecular Ancestry Network (MANET) project traces evolution of protein architecture in biomolecular networks. We describe metabolic MANET, a database that links information in the Structural Classification of Proteins (SCOP), the Kyoto Encyclopedia of Genes and Genomes (KEGG), and phylogenetic reconstructions depicting the evolution of protein fold architecture. Metabolic MANET literally 'paints' the ancestries of enzymes derived from rooted phylogenomic trees directly onto over one hundred metabolic subnetworks, enabling the study of evolutionary patterns at global and local levels. An initial analysis of painted subnetworks reveals widespread enzymatic recruitment and an early origin of amino acid metabolism.

**Conclusion:**

MANET maps evolutionary relationships directly and globally onto biological networks, and can generate and test hypotheses related to evolution of metabolism. We anticipate its use in the study of other networks, such as signaling and other protein-protein interaction networks.

## Background

Cellular metabolism represents a collection of enzymatic reactions and transport processes that convert metabolites into molecules capable of supporting cellular life. It is the best-studied biological network, with highly branched pathways describing the enzymatic processing of metabolites. Though underappreciated, it also represents one of the greatest achievements of science, resulting from almost two centuries of biochemical research.

There is considerable interest in the processes underlying the evolution of cellular metabolism. The existence of a core ensemble of metabolic reactions common to most organisms suggests that the global metabolic structure has been the subject of strong evolutionary constraint. Similarly, network connectivity properties suggest modular components typical of evolved systems [1-3] and emergence of hub metabolites involved in many reactions by enzyme specialization [4]. How metabolic networks function and change as organisms increased in complexity remains an important question, making metabolism an interesting model for the evolution of biomolecular networks.

Metabolism is largely driven by enzymatic specificities. Consequently, the origin and evolution of metabolic networks can be explored advantageously by focusing on protein molecules. However, metabolism is very ancient and parts of the metabolic network probably evolved prior to the origin of cellular life from reactions that could have proceeded without catalysis or with inorganic catalysts [5]. This view is supported to some extent by *in vitro *experiments that try to simulate pre-biotic chemistry. It is likely that polypeptides became metabolic catalysts through takeover of pre-biotic reactions [6]. The earliest enzymes were probably weakly catalytic and multifunctional with broad specificities. Gradually, more numerous, effective, and specific enzymes evolved from the multifunctional enzymes through gene duplication, mutation and divergence. The only condition necessary for such a scenario appears to be selection for faster growth [7].

As enzymatic pathways became more complicated, new enzymatic functions and metabolic pathways could have been generated by recruitment of individual enzymes from the same or different pathways, or by enzymatic recruitments *en masse *from entire pathways. In this regard, several possible scenarios for the evolution of enzymes in metabolic pathways have been proposed [8]. One popular scenario is the "backwards" (or retrograde) evolution hypothesis in which pathways evolve driven by successful production of their end products [9]. Here, biosynthetic pathways undergo retro-evolution, with recruitment of enzymes (from within or outside the pathway) to host sites sequentially more remote from the end product of the pathway. By a symmetrical argument, catabolic pathways could have evolved sequentially from the metabolite being degraded [10]. An alternative scenario is one in which new pathways evolve by "enzyme recruitment" from diverse donor sites throughout metabolism [11]. This hypothesis assumes there is already an active enzymatic core with multifunctional and/or specialized enzymes from which new enzyme recruits are drawn for metabolic innovation. The result is an evolutionary "patchwork" of homologous enzymes that are present in different pathways [6].

There is considerable evidence supporting the patchwork recruitment scenario [8]. For example, enzymes with α/β barrel fold structure that catalyze similar reactions occur across metabolic pathways [12]. These patterns of structural homology appeared to be pervasive when structural assignments and sequence comparisons were used to analyze the small-molecule metabolism in *Escherichia coli *[13,14]. Recruitment occurred with little regularity in these instances. However, proximity of donor and host sites appear to influence the probability of recruitment, with diversification to new host sites occurring mainly from nearby enzymes and varying with metabolite usage and enzyme class [15]. It is noteworthy that sequence comparisons revealed homologous enzyme pairs occurring close to each other in the metabolic network more often than expected by chance [16]. However, these homologous enzyme pairs had similar functions that could be best explained by patchwork recruitment. These observations suggest the retrograde evolution model played a small part in the process of metabolic enzyme evolution. None of these studies however used a phylogenetic approach to establish evolutionary patterns. Because common ancestry is the organizing principle underlying biology, we generate here a database for the evolutionary study of metabolic networks that integrates fragmentary knowledge about molecules and their interaction with phylogenomic information. This database characterizes patterns of evolution in cellular metabolism that will help to resolve the contribution of various plausible evolutionary scenarios.

Proteins consist of domains, compact sections of the protein molecule that have distinct structure, function and evolutionary history [17] and are used as a basis for structural classification [18,19]. Protein domains represent a finite number of folding architectures, the so-called protein folds [20]. These folds are highly diverse and are believed to originate from a common ancestor [20-22]. Crystallographic information gathered by structural genomics has enhanced our knowledge of the universe of fold architecture. This effort has been complemented by matching structures defined by a library of folds to genome sequences. For example, "occurrence analysis" methods compare how often a particular fold or fold group occurs in various genomes [23]. This provides insights on the evolution of genomes because proteins with similar sequences have analogous structures, and structures are highly conserved in nature [23,24]. Using this approach, whole-genome trees were reconstructed based on the occurrence of fold architectures and gene orthologs in genomes. These trees resemble those reconstructed from the sequence of the small subunit of ribosomal RNA (rRNA) and showed the tripartite nature of our organismal world [24-29].

We recently used an approach based on a census of folds to study protein diversification and reconstruct universal phylogenomic trees describing the evolution of protein fold architecture [27,30,31]. Our approach is based on two fundamental premises: (1) that protein structure is far more conserved than sequence and consequently carries considerable phylogenetic signal, and (2) that protein folds that are successful and popular in nature are generally more ancestral. Trees reconstructed from global fold-usage statistics showed there were clear evolutionary patterns in the appearance of protein folds. For example, the folds in the α/β protein class appeared at the base of the tree, and were followed by those in the α+β, all-α, all-β, small, and multidomain classes respectively [27]. A similar tendency was recently observed when reconstructing parsimonious scenarios describing occurrence patterns of folds in organismal phylogenies [32]. We also traced the number of enzymatic functions associated with folds in the tree of protein architecture and found that older folds were associated with an increased number of enzymatic functions. These phylogenomic studies suggest enzymatic multifunctionality was replaced by specialized function during evolution. Interestingly, a direct association between protein classes and function was previously revealed in which for example α/β folds were disproportionately associated with enzymes, especially transferases and hydrolases, while all-α and small proteins were associated with non-enzymes [33]. Within α/β folds, five folds were the most functionally versatile (TIM-barrel, Rossmann, ferredoxin, α/β hydrolase, and P-loop NTP hydrolase). These folds were all placed at the base of our phylogenomic tree.

**Figure 1:**
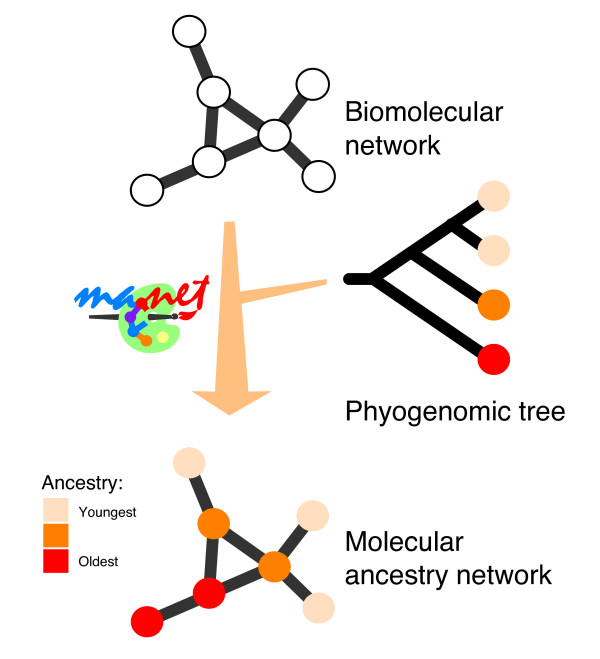
**The Molecular Ancestry Network (MANET)**. The MANET project traces evolutionary patterns derived from phylogenomic analysis along biomolecular networks. In one implementation of our method, we "paint" network nodes with a color scale that reflect their ancestries.

The Molecular Ancestry Network (MANET) project uses information embedded in our phylogenomic trees to trace evolution of protein fold architecture in biomolecular networks (Fig. 1). In this paper, we describe the construction of metabolic MANET, a database that explores the evolution of modern metabolism. Metabolic MANET uncovers evolutionary patterns in metabolism at global and local levels and reveals evolutionary relationships between protein architecture and enzymatic function. We used Protein Data Bank (PDB) entries to link enzymes to protein folds and hidden Markov models (HMMs) to assign structures to enzymes for which there was only gene information. Phylogenomic trees were then reconstructed from protein fold occurrence in sequenced genomes representing species within the three organismal domains of life. These trees were used to assign a relative age (ancestry) to each metabolic enzyme for which a protein structure was known or could be inferred. Finally, ancestries were "painted" onto metabolic subnetwork representations with a color (value) that described the relative age of each enzyme in metabolism.

## Construction and content

### Approach

**Figure 2:**
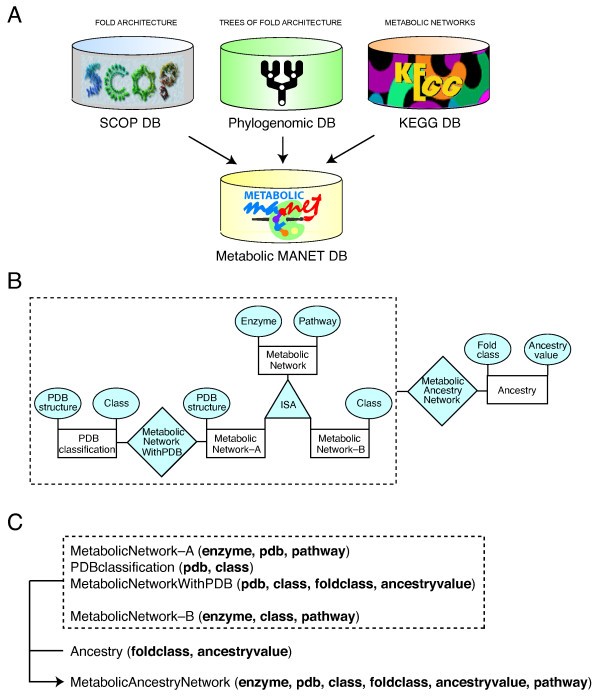
**Principles of database design**. A. The metabolic MANET database links the KEGG database, the SCOP database, and a database of phylogenomic trees of protein fold architecture. B. Entity-Relationship (ER) data model. "MetabolicAncestryNetwork" is the union of the relation "MetabolicNetworkwithPDB and Ancestry" and the relation "MetabolicNetwork-B and Ancestry". C. Relation schema for the ER model, specifying the name of each file and defining "MetabolicNetwork-A" and "MetabolicNetwork-B" (see Additional file 1).

MANET links three flat files describing the metabolic pathways database of the Kyoto Encyclopedia of Genes and Genomes (KEGG), the Structural Classification of Proteins (SCOP) database, and phylogenomic trees reconstructed from a genome census of protein folds (Fig. 2A). KEGG provides integrated information about cellular metabolism [34,35]. This database contains graphical diagrams describing 132 metabolic subnetworks grouped into 11 mesonetworks. Mesonetworks pool subnetworks with functionally-related pathways. For example, the mesonetwork "amino acid metabolism" includes 16 subnetworks that describe the synthesis and degradation of specific amino acids. The KEGG pathway file included 4,362 metabolic enzymes classified according to their function, 137 pathways and molecular complexes, 12,778 PDB structural entries, and nucleotide and amino acid gene sequences. SCOP maps Protein Data Bank (PDB) entries onto a structural classification of proteins [18]. The SCOP file included a classification of 24,037 PDB entries associated with fold families, fold superfamilies, and folds. The phylogenomic trees describe phylogenetic relationships of protein fold architectures and were reconstructed from fold occurrence in genomes that have been completely sequenced. The phylogenomic file summarizes the evolution of 784 folds in 174 genomes. Figure 2B illustrates the data model of MANET in terms of entities and their relationships. The schema shown in Figure 2C specifies the column heads for the entities and relationships. MANET links all components of the schema relating protein folds and enzymatic function within an evolutionary perspective.

### Phylogenetic analysis

A set of phylogenetic features (characters) was used to describe the frequencies with which individual protein folds occur in an individual genome. This frequency was termed *genomic abundance *(*G*). At global levels, it describes how popular is a fold architecture in nature. In order to calculate *G*, we assigned structural domains to proteins. We did this using the HMM-based SUPERFAMILY database [36]. SUPERFAMILY assigns protein structures to amino acid sequences at the fold superfamily level, i.e. at a hierarchical level capable of pooling proteins for which there is structural and sequence evidence of a common evolutionary ancestor [37]. The HMM searching protocol uses a probability cut-off *E *of 0.02. Differences in topologies of trees reconstructed with more stringent cut-off values were found negligible [29], so we did not explore the role of this parameter. We also used the hierarchical scheme of SCOP to assign superfamilies to folds. Version 1.67 classifies 65,122 domains present in 24,037 PDB entries into 1,447 superfamilies and 887 protein folds. We analyzed the genome sequence of 36, 19 and 117 genomes from Eucarya, Archaea, and Bacteria, respectively, and reconstructed phylogenetic trees describing the evolution of 784 protein folds. Phylogenomic characters were coded as previously described [27]. *G *was normalized using gap-recoding techniques to compensate for differences in genome size and proteome representation, and was then subjected to logarithmic transformation to account for unequal variances. The data was range standardized to a 0–20 scale compatible with most phylogenetic analysis programs, treated as linearly ordered multistate characters using an alphanumeric format with numbers 0–9 and letters A-K, and encoded in the NEXUS format. The ANCSTATES command was used to polarize characters assuming that the number of protein representatives in a genome exhibiting a particular fold increases in the course of evolution. Character argumentation is supported by model and assumptions described and discussed previously [27,30,31]. Phylogenies were reconstructed using maximum parsimony (MP) as the optimality criterion in PAUP* [38], and phylogenetic reliability was evaluated by the bootstrap method [39]. Optimal most-parsimonious trees were obtained from heuristic maximum parsimony searches with tree-bisection-reconnection (TBR) branch swapping and 100 replicates of random addition sequence after exclusion of uninformative phylogenomic characters. To decrease search times during branch swapping of suboptimal trees, not more than one tree was saved in each replicate. The structure of phylogenetic signal in the data was tested by the skewness (*g*_1_) of the length distribution of > 10^4 ^random trees and permutation tail probability (PTP) tests of cladistic covariation using >10^3 ^replicates. Homoplasy and synapomorphy were measured with ensemble consistency (CI) and retention (RI) indices.

Because reconstructed trees were intrinsically rooted, we established the relative age of individual protein folds by measuring a distance in nodes from the hypothetical ancestral fold on a relative 0–1 scale. Concretely, we counted the number of nodes in every lineage from the root to the terminals of the tree and divided this number by the maximum number of nodes in a lineage. This *node distance *(*nd*) depicts the number of cladogenic events along a lineage and was used as an indicator of the *ancestry *of each metabolic enzyme for which a protein structure is known or could be inferred. A Perl script was written to extract ancestry values from phylogenetic trees.

Note that the central assumption that folds are more prevalent and widely shared the more ancient is their origin follows a parsimony rationale supported by patterns of distribution and sharing of protein folds across life, statistical analyses, and phylogenetic considerations [27]. Our model is global and applies to the world of genomes, sampled by phylogenetic characters describing organisms in the three domains of life. Consequently, the model should be relatively insensitive to "genome lineage" specific factors such as selection pressures for genome expansion or reduction, life styles of organisms considered, and horizontal gene transfer events. However, our model is minimalist in that it does not account for differences in evolutionary rates across lineages and changes in the size of the protein world expected to have occurred during evolution. In particular, evolution of individual folds may be influenced by factors affecting rates, induced for example by instrinsic fold properties (e.g. flexibility). Consequently, fold ancestries should be regarded as lower bounds for times of origin.

### Parsing

We retrieved flat files directly from KEGG [40] and SCOP [41]. The retrieval date for data presented in this paper was December 2004. We used Perl scripts to parse data from the files, store the parsed data into the entities of MANET, and manipulate the collected information in the database system. For example, we parsed data fields such as enzyme entry, pathway, and structural PDB entry from the database file "enzyme" obtained from KEGG, and inserted them into the entity called "MetabolicNetwork-A". Similarly, we parsed PDB entries (structures) and SCOP protein classifiers from database files such as "dir.cla.scop.txt_1.67" obtained from SCOP into "PDBclassification". Ancestry values were recorded into the entity "Ancestry".

### Join operations and coloring of enzymes

Join operations necessary to combine information from entities in MANET and coloring of enzymes are described in the supplementary data [see Additional file 1]. The join operation linked 687 enzymes to protein folds. This represents about 35% of total enzymes associated with pathway information in the KEGG database. Coloring depicts graphically the relative age of metabolic enzymes, when these are associated to folds. A 'code generator' was used to output PHP and Visual Basic script files that paint the ancestries of enzymes on metabolic subnetwork diagrams.

### Superfamily prediction using HMMs

In order to increase protein fold assignments to enzymes in metabolic pathways, we used a library of HMMs for remote homology detection in SUPERFAMILY. We used the model library, Perl wrapper scripts for sequence alignment, and the Sequence Alignment and Modeling System (SAM), and ran the SUPERFAMILY software package locally in a 15-node dual-processor Xserve cluster using the genes catalog file obtained from KEGG. This file includes amino acid and nucleotide sequence information from complete or partial genome sequences. Details can be found in the supplementary data file.

### Statistical analysis

HMM-based structural prediction increased assignments of protein fold to enzymes in metabolic pathways. However, biases in fold superfamily prediction could affect evolutionary tracings in networks. To test the effect of prediction bias, we selected amino acid sequences associated with enzymes that had structural PDB entries. Statistical tests were performed using the SAS software package [42]. The frequency distributions of ancestries derived directly from structural models (group A) and from HMM prediction (group B) were compared by using the Wilcoxon rank sum test. This test is one of several statistical tests that analyze two groups. It requires equal variances and independence of samples taken at random, but unlike t-based tests, it does not require for groups to have normal distributions [43]. The null hypothesis of no difference between groups was tested using the NPAR1WAY procedure in SAS with grouped data.

We also performed a global statistical analysis of distribution of ancestries in mesonetworks. All average ancestry values of enzymes in the 11 mesonetworks described in KEGG were analyzed by ANOVA and by multiple pair-wise comparisons with the SAS package. There is a high probability of declaring at least one pair of means significantly different when running multiple comparisons unless the per-comparison error rate α is small among sample means [43]. We therefore considered Type I error rates related to multiple comparisons, choosing the Least Squares Means with adjustment for multiple comparisons as the general linear model (GLM) procedure and the Tukey-Kramer method as the post-hoc test. The Tukey-Kramer multiple-comparison procedure controls error rates by testing every pair of means. The mesonetwork distribution data was subjected to logarithmic [log (y+1)] transformation. This reduced variances by about 1% and approximated data to normality.

## Utility

A substantial body of literature has shown that linking protein structure to proteins in biomolecular networks can be advantageous. Some of these studies involved comprehensive structure-enzyme mapping exercises and explored mechanistic aspects related to enzymatic function. For example, global analysis of small-molecule metabolic pathways in *Escherichia coli *has shown extended distribution of structural homologues across metabolism [13,14,44,45], sometimes confined to specific subnetworks [45]. This and other evidence suggests the presence of widespread enzymatic recruitment and other evolutionary processes. Linking structure to metabolic function has also shown that in metabolism, catalytic mechanisms and co-factor binding properties are conserved while substrate specificity is variable [14,40]. It appears it is easier to evolve binding sites than catalytic mechanisms. A recent study also shows for example that only a few fold superfamilies exhibit great substrate diversity, while most do not [46]. Knowledge of structure can therefore help generate hypotheses about possible substrates associated with a protein.

However, proteins with similar structures may or may not be evolutionary related, and other approaches that introduce phylogenetic views are therefore needed. For example, Copley and Bork [12] used sequence, structure, and function to derive a phylogeny describing the evolution of members of 12 superfamilies with α/β barrel fold structure involved in metabolism. Establishing homologies at these levels provided indications that these fold superfamilies shared a common origin.

Our metabolic MANET database adds an evolutionary component to global assignment of protein structure to enzymes in metabolic subnetworks. Phylogenomic trees that describe the evolutionary relationship between protein fold architectures were used to define ancestries of individual folds, and these were traced onto metabolic subnetwork diagrams representing modern metabolism. Because our phylogenies were reconstructed from fold occurrence in hundreds of organisms with fully sequenced genomes spanning all three domains of life, they represent global phylogenetic statements about the protein world [27]. These statements are therefore appropriately mapped to enzymatic structure in global cross-organismal representations of metabolism, such as those embedded in KEGG.

Tracing evolution of protein architecture in metabolic networks is useful. The exercise can uncover evolutionary patterns of architectural diversification within individual pathways of a subnetwork or between subnetworks and mesonetworks. Because metabolism is highly conserved, with about half of enzymes present in at least one species from the three domains of life [47], it is therefore possible to identify evolutionary patterns unique to the metabolic core that is universally conserved. MANET can also help identify processes driving the evolution of modern metabolism at local and global levels, including enzyme recruitment. It also allows query of SCOP, PDB, Enzyme Commission (EC) numbers, and subnetwork information that is useful for discovery of links between enzymatic activities and structures.

The web interface of MANET implements server-side scripts with a system of database management that provides visualization, query, and statistical analysis dynamically. All components of MANET are Perl-based and easily updated. Enzymes associated with protein folds are painted directly on subnetwork diagrams. Each enzyme is also hyper-linked to the KEGG database so that the user can retrieve additional information by clicking in the diagram. MANET also provides numerous functionalities, which enable searching for specific protein folds with defined ancestry values, displaying the distribution of enzymes that are painted, and exploring folds in individual subnetworks. Finally, the frequency distribution of ancestry values for each subnetwork can also be visualized.

## Results and discussion

**Figure 3:**
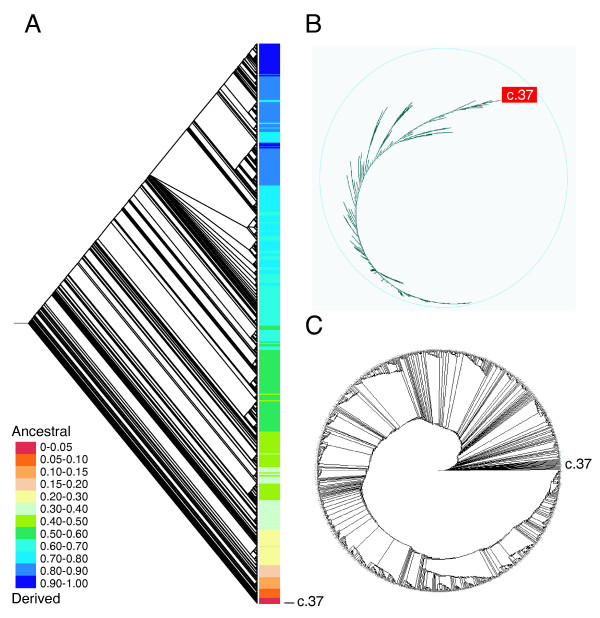
**Phylogenomic tree reconstruction of protein fold architecture generated from a domain census in 174 completely sequenced genomes**. The structural census was defined by advanced HMMs and assigned domain structure to about 60% of genomic sequences. Three optimal trees of 115,818 steps were obtained after a heuristic search (CI = 0.134, RI = 0.696; RC = 0.093; g_1 _= -0.406; *p *< 0.01). The consensus phylogenomic tree is shown as a rooted dendrogram with terminals colored according to ranges of ancestry values (A), an unrooted hyperbolic tree (B), and a rooted circle tree (C). Terminal taxa are not labeled except for the fold of oldest origin, c.37, the P-loop hydrolase fold.

**Figure 4:**
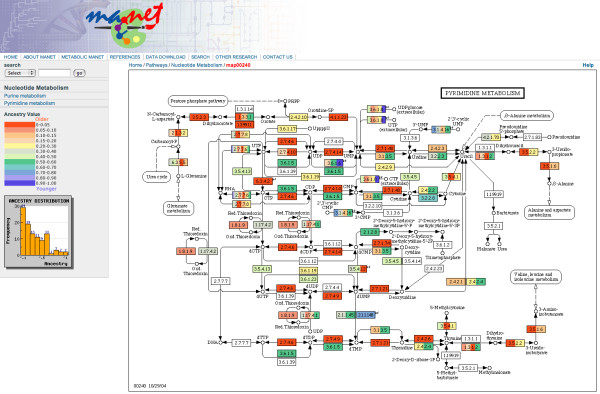
**Representative subnetwork diagram describing molecular ancestries in metabolic MANET**. A colored scale is use to assign binned ancestry values to enzyme nodes named with EC numbers. The red color represent enzyme nodes with the oldest ancestry (i.e. with ancestry values falling within the oldest range) and the blue color those of youngest ancestry. Some enzymes have more than one structural assignment. They are multidomain proteins or have different structures in different organisms. Colored enzymes with ancestry assignments resulting from HMM-based predictions are distinguished with a "sf" marking at the top right of rectangles depicting enzymatic nodes. Each subnetwork diagram also shows a frequency distribution plot of ancestries.

The MANET database project traces the evolution of protein structure in biomolecular networks with bioinformatic, phylogenetic, and statistical methods. Metabolic MANET links the SCOP and KEGG databases to universal phylogenies of protein fold architecture. The database was assembled in multiple steps. We first reconstructed phylogenomic trees describing the evolution of protein folds in 174 proteomes belonging to Eucarya, Archaea and Bacteria. These trees are large and can be visualized using hyperbolic tree visualization tools [48]. Figure 3 shows cladogram, hyperbolic, and circular tree representations of the tree of protein architecture used in this study. The tree is consistent with phylogenies generated previously from a set of 32 proteomes using the same approach [27]. These tree reconstructions were then used to assign a relative age (ancestry) to each fold based on how many cladogenic events occurred in each lineage (Fig. 3). Finally, ancestries were literally painted onto metabolic subnetworks with information derived from SCOP, KEGG and HMM-based fold superfamily prediction tools. Figure 4 describes a representative subnetwork of metabolic MANET showing enzymatic nodes painted with molecular ancestries. Please note that ancestries represent a lower limit on the time at which the fold might have been adopted for a particular enzymatic activity.

Reconstructed trees were based on a genomic census of protein architecture. Consequently, they depend on the accuracy of genomic databases, a balanced genomic sampling of the living world, efficient and accurate assignment of structures to proteins, a structural classification scheme that depicts evolutionary patterns, and methods of phylogenetic tree and character state reconstruction. The influence of these factors has been discussed previously [27,30,31]. While there is no possible gold standard that can be used to confirm the validy of phylogenomic statements, the genome census data we use to generate the tree of fold architectures was also used to generate trees of proteomes, and these trees group organisms in the three domains for the most part according to established organismal classification [Wang and Caetano-Anollés, ms. in preparation]. This observation supports the validity of phylogenetic signal embedded in the data.

Our study also rests on the accuracy of SCOP, a robust protein classification scheme [18,49], and on the monophyletic nature of protein folds and superfamilies. Consequently our inferences should be regarded as rough first approximations. While we do not expect major changes in the operational definition of a protein fold, many folds could be better described by "continuous" rather than "discrete" distributions in structure space [50]. Furthermore, we trust SCOP hierarchies reflect true evolutionary groupings. In SCOP, proteins in families express clear evolutionary relationships. They generally exhibit >30% pairwise residue identities or have functions and/or structures that provide definite evidence of common descent. Similarly, fold superfamilies contain proteins with structural and functional features that are highly suggestive of a common evolutionary origin. However, highly popular folds encompass collections of fold superfamilies that share the same arrangement and topology of secondary structures but may not have a common evolutionary origin. Consequently, the monophyletic nature of protein folds needs to be examined case by case, as has been done for the (βα)_8 _barrels [12,51].

**Figure 5:**
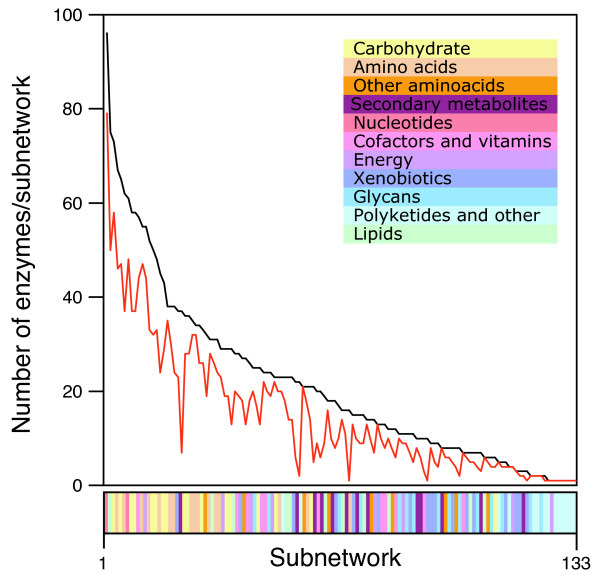
**Painting efficiency in metabolic subnetworks**. The plot describes the total number of enzymes (black line) and the total number of painted enzymes (red line) in each of the 132 subnetworks described in KEGG, sorted according to enzyme number. Subnetworks belonging to individual mesonetworks are identified with different colors.

Currently, metabolic MANET contains 23,217 entries linking 1,255 enzymatic activities to PDB entries, folds, ancestry values, and pathways. A total of 6,552 PDB entries are associated with metabolic subnetworks. Based on information derived mostly from crystallographic structural models, 33% of metabolic protein nodes were painted in phylogenetic tracings of the metabolic pathways that are registered in KEGG. Use of HMMs that assign probable fold superfamily identities to protein sequences increased the fraction of painted enzymes to 63%. Individual steps in the analysis and sorting of data can be found in the supplementary data [see Additional file 1]. Among the 132 subnetworks from the MANET database, 122 subnetworks described metabolic pathways and 10 subnetworks described processing of genetic, environmental and cellular information. On average, 72% of enzymes were painted in metabolic MANET [see Additional file 1], ranging from 6% for the monoterpenoid biosynthesis subnetwork to 100% for subnetworks such as aminoacyl-tRNA biosynthesis, reductive carboxylate cycle (CO_2 _fixation), and novobiocin biosynthesis. Large subnetworks such as those belonging to nucleotide, carbohydrate and amino acid mesonetworks were painted similarly to others. Interestingly, some subnetworks contained more evolutionary information. Subnetworks such as purine metabolism and pyrimidine metabolism that contain many more enzymes than others had about 83% and 79% of enzymes painted, respectively. Only 10 subnetworks (7.6%) in metabolic MANET did not have entries associated with ancestry values. These were beta-lactam resistance and clavulanic acid biosynthesis in mesonetwork "biosynthesis of secondary metabolites", 1,1,1-Trichloro-2,2-bis(4-chlorophenyl) ethane (DDT) degradation and bisphenol A degradation in "biodegradation of xenobiotics", glycosylphosphatidylinositol(GPI)-anchor biosynthesis in "glycan biosynthesis and metabolism", and biosynthesis of ansamycins, biosynthesis of siderophore group nonribosomal peptides, biosynthesis of vancomycin group antibiotics, and biosynthesis of type II polyketide products in mesonetwork "biosynthesis of polyketides and nonribosomal peptides". The efficiency of painting was not biased by subnetwork size (Fig. 5).

**Figure 6:**
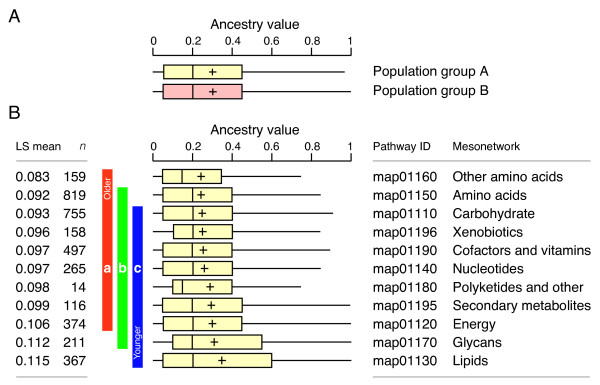
**Box-and-whiskers plot describing global frequency distribution profiles**. A. Comparison of ancestry values derived from structural models using the join operation (population group A) and predicted using HMMs (population group B) in metabolic MANET. B. Statistical analysis of ancestry distribution in metabolic mesonetworks. Mesonetwork distributions next to vertical bars headed by the same letter are not significantly different (*p *< 0.05). The Least Squares (LS) means and number of enzymes analyzed (*n*) are given for each mesonetwork. Skeletal boxes describe median, lower and upper quartiles, and whiskers describe maximum and minimum values. Crosses indicate mean values.

Evolutionary tracing in MANET reflects information derived from structural models present in the PDB or represents HMM-based inferences of structural classification. In order to test if biases in fold superfamily predictions could affect evolutionary tracings in networks, we designed a statistical test that compared frequency distributions of ancestries derived from the join operation defined by structural models (population group A) or derived from HMM-based predictions (population group B). We selected amino acid sequences associated with enzymes that had structural PDB entries and participated in the join operation. A total of 72,354 amino acid sequences within this category were selected, and resulting ancestry values were calculated and analyzed (Fig. 6A). The mean (± SE) for ancestry value distributions was 0.277 ± 0.008 and 0.296 ± 0.006 for populations groups A and B, respectively. Basic statistical parameters showed both ancestry frequency distributions were not normally distributed but had the same shape with almost the same variance (0.072 and 0.078 for groups A and B). However, measurements of skewness (1.068 and 0.990) and kurtosis (0.233 and -0.029) indicate the distribution of group B was shifted to the right of the distribution of group A. The Wilcoxon rank sum test showed that the *p*-value (0.0553) for a one-tailed test was greater than the expected value for α = 0.05 (using both normal or t-approximation), failing to reject the null hypothesis that ancestry values distributions for groups A and B were identical. We therefore conclude that ancestry value distributions derived from structural models or HMM predictions were not significantly different at the 95% confidence level.

We also tested the accuracy of the HMM prediction. We selected PDB entries corresponding to enzymes in KEGG that participated in the join operation and were classified structurally, assigned PDB sequence records downloaded from ASTRAL [52] to the PDB entries, and analyzed the PDB sequences using the HMM package at E value = 0.02. Superfamily IDs and structural classifications corresponding to the PDB sequences were retrieved. Out of 21,173 PDB entries corresponding to enzymes identified by the join operation, 20,941 PDB entries mapped to ASTRAL. Sequences corresponding to these PDB entries were analyzed further. The HMM-based method rejected 212 sequences, leaving a total of 20,729 PDB entries with an assigned fold structure. Out of these, only 67 PDB entries differed in the expected fold assignment. At the fold superfamily level of classification, 106 PDB entries differed in the expected superfamily assignment. These results indicate that the HMM-based superfamily prediction can be performed at 98% accuracy levels. The details of this analysis can be found in our website.

The assignment of numerical ancestry values to enzymes in cellular metabolism uncovers evolutionary patterns of architectural diversification within the metabolic network. A quick examination of ancestry distributions depicted in each subnetwork and mesonetwork diagram of the MANET database reveals that enzymes of old origin generally coexist with those of recent origin (see example subnetwork; Fig. 4). A more detailed analysis of individual subnetwork paintings reveals the absence of clear patterns in individual pathways. Enzymes of old origin were generally followed haphazardly by enzymes of recent origin, and vice versa, with no apparent pattern along pathways. The patchy appearance of ancestries in subnetworks belonging to all metabolic mesonetworks supports strongly the enzyme recruitment (patchwork) evolutionary scenario as the major evolutionary force responsible of present day metabolism. Metabolic MANET makes visually evident enzymatic recruitment patterns that have been observed previously (*e.g*. [13,14]), placing them into a relative evolutionary time frame. This offers the possibility of reconstructing temporal timelines of recruitment episodes in subnetworks and mesonetworks. Other evolutionary alternatives (backward evolution, forward evolution, de novo invention, pathway duplication, etc.) are not readily visible in our evolutionary tracing exercise. A detailed analysis of each subnetwork will be required to reveal the incidence of these possible evolutionary mechanisms. Pathway 'take-over' mechanisms in which new enzymes replace either pre-biotic chemistries or old enzymes, and 'co-option' mechanisms in which old enzymes gain novel functions, are also possible. In this regard, we are currently evaluating possible take-over episodes in metabolic subnetworks that may result from *en masse *enzymatic recruitment processes occurring in subnetwork pathways. We envision that uncovering take-over patterns in MANET at global levels will require extensive information about possible pre-biotic chemistries and novel phylogenetic approaches.

The evolutionary patterns revealed by MANET have other interesting implications. If we assume that pre-biotic chemistries remain imprinted in modern metabolism as relics of the pre-biotic world, patterns of enzymatic ancestries may reveal fundamental steps in prebiotic evolution. These evolutionary patterns may still manifest in the subnetworks despite obscuring events such as take-overs. Morowitz [53,54] proposed that metabolism evolved through the sequential addition of shells to an "energy amphiphile" core (shell A), which consisted of the Krebs cycle, glycolysis, and fatty acid biosynthesis. The amination of 2-ketoglutarate was the gateway to shell B, the synthesis of most amino acids. In shell C sulfur was incorporated into cysteine and methionine. The gateways to shell D, ring closure and synthesis of nitrogen and dinitrogen heterocycles, gave access to purines, pyrimidines, and many cofactors, including B_12_. This scenario suggests that compounds in shell D evolved after enzymes (derived from shell B and C) and were not a part of prebiotic chemistry. The energy amphiphile core is consistent with Wächtershäuser's proposal that life evolve on pyrite (see [5]). According to this theory of an iron-sulfur world, a reductive citric acid cycle that used thio-organic homologues evolved early and was later coopted for oxidation. The reductive citric acid cycle, an autocatalytic network, expanded by branch reactions into higher homologous cycles. This archaic network included pathways for the synthesis and degradation of phosphorylated sugars, some amino acids (glutamate, aspartate, alanine, lysine), fatty acids and isoprenoids, coenzymes (including tetrapyrroles), and purines.

When ancestry patterns embedded in the subnetworks of MANET were analyzed, sequential evolution of metabolic "shells" was not obvious. However, pervasive enzyme recruitment could have masked the original pre-biotic evolutionary patterns. In fact, we performed a global statistical analysis of the distribution of ancestries of enzymes in metabolism, testing if global evolutionary patterns in metabolism matched possible "shell" scenarios (Fig. 6B). We calculated mean ancestry levels from frequency distribution patterns of ancestry data for mesonetworks, assuming these values were indicative of an average age of the enzymes examined. The statistics of distribution of ancestries in mesonetworks showed that distributions differed significantly in mean ancestry levels (*p *< 0.0001; ANOVA, F-test). Furthermore, the analysis revealed that amino acid mesonetworks were the oldest and lipid (including steroid) and glycan mesonetworks were relatively recent evolutionary additions (*p *< 0.05; Tukey-Kramer multiple comparison). The early evolutionary appearance of mesonetworks related to amino acid metabolism suggests that metabolic routes leading to the synthesis of polypeptides (shells B and C of Morowitz) 'internalized' early into the protein-based enzymatic machinery.

While mesonetworks may pool subnetworks of different average ancestry complicating interpretation, our results are nevertheless consistent with the shell hypothesis of Morowitz [54]. In this regard, the early evolution of amino acid metabolic mesonetworks raises an interesting question. Why were the energy amphiphile core pre-biotic functions not the first to be replaced by enzymatic counterparts? These pre-biotic functions were the oldest and probably the most stable. One explanation is that replacement of non-enzymatic amino acid metabolic pathways follows the need to secure amino acid synthesis for protein-based enzymatic activities. It is possible that pre-biotic entities could have competed with each other for environmental resources during this early stage of metabolic evolution. Within this context, the opening of the gateway to amino acid synthesis proposed by Morowitz could have offered the possibility of creating enzymes that would perform pre-biotic functions more effectively.

## Conclusion

We constructed a database that links biomolecular networks, protein structure, and phylogenomics. Metabolic MANET traces the evolution of protein structure directly onto metabolic networks defined by KEGG, enabling the study of evolutionary patterns in metabolism at global and local levels. MANET can be a valuable resource and constitutes a discovery tool. Individual pathways within subnetworks and mesonetworks can be examined and evolutionary patterns can be detected by visual inspection or statistical analysis of ancestry distributions. The database has many possible applications. For example, it can be used to search for patterns of fold superfamily sharing between subnetworks, with the aim of displaying the coordinated evolution of the subnetworks. Evolutionary information deposited in metabolic MANET will be enhanced by the exponential increase in the number of genomes that have been sequenced, the number of fold architectures uncovered, and the number of metabolic enzymes with gene assignments. The principles used in the construction of metabolic MANET are general and can be extended to other biomolecular networks. In the near future, we plan to analyze other networks of importance, such as cell signaling and other protein interaction networks.

## Availability and requirements

The MANET database can be accessed via the Internet at . Data materials are formatted as EXCEL or column-delimited flat files for parsing with programming tools such as Perl and are openly available at our web site. The use of information obtained from the KEGG and SCOP databases is restricted by licensing conditions specified elsewhere. Contact information: THE MANET PROJECT, Atelier of Plant Bioinformatics, E-mail: evolutionary-manet@uiuc.edu.

## Authors' contributions

HSK acquired data, developed methods, wrote the codes, created the database, and performed statistical analyses. GCA and JEM initiated and conceived the project. GCA guided HSK in research and interpretation of results. GCA and HSK created figures and did most of the writing with input from JEM. All authors read and approved the final manuscript.

## Supplementary Material

Additional File 1 Description of join operations, superfamily prediction using HMMs, coloring, and analysis and sorting of data. Table 1 describes painting efficiency in metabolic MANET. Click here for file
