# Supplementary material for: MANET: tracing evolution of protein architecture in metabolic networks
Source: BMC Bioinformatics. 2006 Jul 19;7:351. doi: 10.1186/1471-2105-7-351 (PMC1559654; doi:10.1186/1471-2105-7-351)
Supplement: Additional File 1 — Description of join operations, superfamily prediction using HMMs, coloring, and analysis and sorting of data. Table 1 describes painting efficiency in metabolic MANET. [file 1471-2105-7-351-S1.pdf]

SUPPLEMENTARY DATA

MANET: tracing evolution of protein architecture in metabolic networks

Hee Shin Kim[1], Jay E. Mittenthal[2], Gustavo Caetano-Anollés[1§]

[1]Department of Crop Sciences, and [2]Department of Cell and Developmental Biology, University of Illinois, Urbana, IL 61801, USA

[§]Corresponding author

Join operations

We combined information from two or more entities in MANET with the join operation, extending information about the individual entities being considered. For example, we joined "PDBclassification" and "MetabolicNetwork-A" together using properties common to both entities (Fig. S1A) and obtained a record set for the new entity "MetabolicNetworkWithPDB", which describes protein fold(s) in enzymes of subnetworks. Similarly, we joined the "MetabolicNetworkWithPDB" and the "Ancestry" entities together using structural PDB entries (Fig. S1B), obtaining a record set for "MetabolicAncestryNetwork" that included the mapping of protein folds and ancestries to enzymes in subnetworks (Fig. S1C). The join operation linked 687 enzymes to protein folds. This represents about 35% of total enzymes associated with pathway information in the KEGG database.

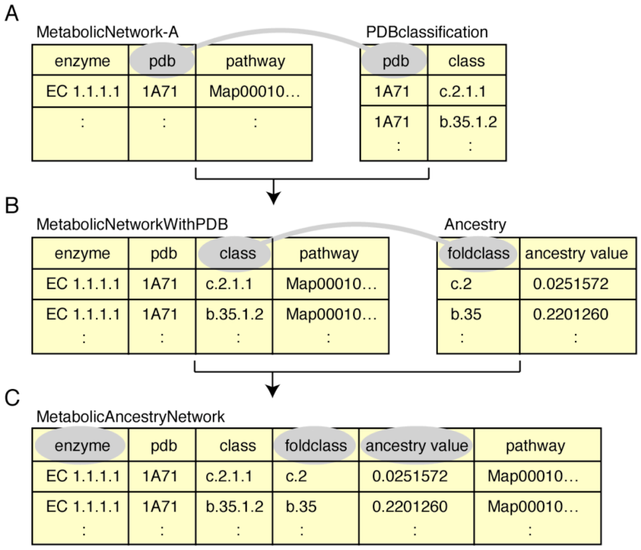

Fig. S1. Join operations in metabolic MANET. The pdb is a common field that links enzymes in "MetabolicNetwork-A" to SCOP assignments in "PDBclassification" (A), creating the record set for the entity "MetabolicNetworkWithPDB" (B). Classification of fold architectures can then join "MetabolicNetworkWithPDB" with "AncestryValue" (B), creating "MetabolicAncestryNetwork" (C), a file that combines enzyme, fold class, and ancestry value together.

Superfamily prediction using HMMs

In order to increase protein fold assignments to enzymes in metabolic pathways, we used a library of HMMs for remote homology detection in SUPERFAMILY. We used the model library, Perl wrapper scripts for sequence alignment, and the Sequence Alignment and Modeling System (SAM) (http://www.cse.ucsc.edu/research/compbio/sam.html), and ran the SUPERFAMILY software package locally in a 15-node dual-processor Xserve cluster using the genes catalog file obtained from KEEG (ftp://ftp.genome.ad.jp /pub/kegg/tarfiles/genes.tar.gz). This file includes amino acid and nucleotide sequence information from complete or partial genome sequences. As shown in Figure S2A, we constructed a database with the amino acid sequences from KEGG, and used Perl scripts to select sequences associated with the enzymes that had gene assignments but lacked structural PDB information. A total of 20,948 amino acid sequences were selected. We applied the same procedure to enzymes that had gene and structural PDB information but failed to join with the structural SCOP classification. In this case, 5,337 additional amino acid sequences were selected. We distributed sequences into individual compute nodes running the HMM package with a threshold *E* value of 0.02 and retrieved superfamily IDs that were associated with targeted enzymes. We also retrieved structural classifications corresponding to the superfamily IDs using the SCOP database file, and inserted them into the entity "MetabolicNetwork-B" of the metabolic ancestry network. Joining the "MetabolicNetwork-B" and "Ancestry" entities together resulted in an additional record set defining the entity "MetabolicAncetryNetwork". These operations increased the number of enzymes linked to protein folds.

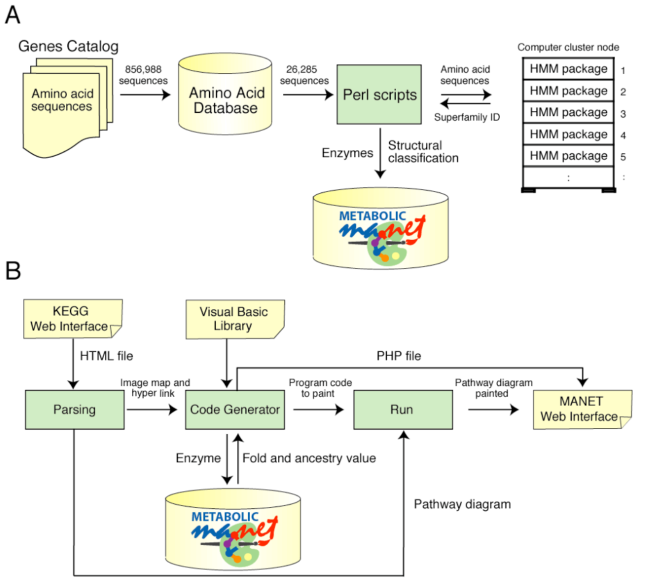

Fig. S2. HMM-based structural prediction and enzyme coloring schemes. A. Perl scripts retrieved 26,285 amino acid sequences related to enzymes identified by the join operation. HMMs were then used to assign superfamily IDs to sequences using a computer cluster. Enzymes with fold superfamily assignments were finally inserted into "MetabolicNetwork-B" in metabolic MANET. B. The Code Generator outputs PHP files that run on the MANET web server and Visual Basic script files that paint with color those nodes that have enzyme entries in subnetwork diagrams.

Coloring

Coloring is a feature that describes graphically the relative age of a metabolic enzyme in the network. The enzymes in MANET are linked to protein fold(s) to which individual ancestries can be assigned and painted on subnetwork diagrams. To accomplish this, we divided the range of ancestries into 12 classes, giving them individual hues in a color scale. We also developed a 'code generator' that created programming codes that paint the colors corresponding to fold ancestry values on pathway diagrams and PHP files that were used for the web interface of MANET. The code generator was run with the HTML source files and pathway diagrams of the KEGG web interface. Figure S2B describes the computerized procedure for coloring. We first downloaded the KEGG web interface including HTML source files and pathway diagrams. We ran the code generator with the HTML source files. The code generator parsed image maps and hyper links from the files, and created the PHP files and programming codes based on a

Visual Basic library. We then ran the programming code with the pathway diagrams on a Windows operating system. As a result, we obtained new pathway diagrams painted with ancestry values.

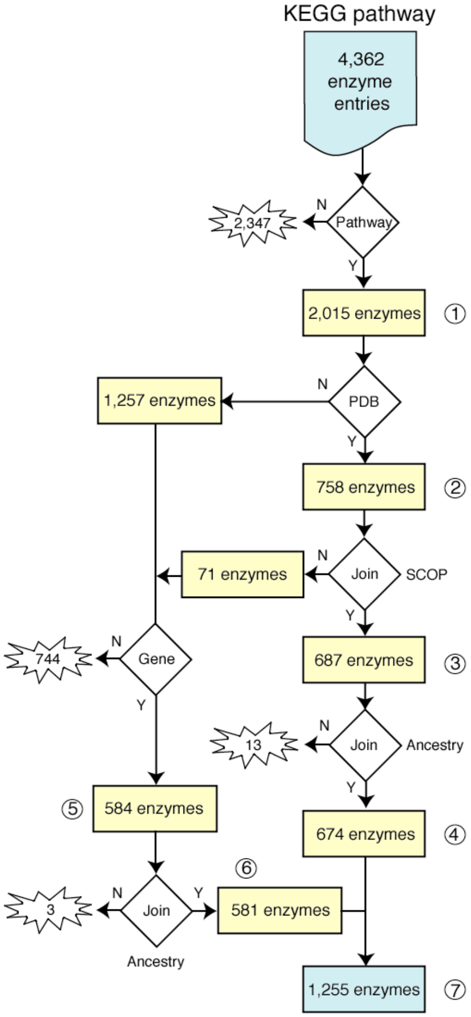

Fig. S3. Analysis and sorting of metabolic MANET data. A total of 4,362 enzyme entries were retrieved from KEGG, 2,015 of which had subnetwork information (①). Among these, 758 enzymes had structural PDB entries associated with SCOP (②), 687 of which could be successfully linked to protein folds (③) and 674 of which could be painted with ancestry values (④). The remaining 1,257 enzymes with no associated PDB entries and the 71 enzymes that could not be assigned to folds were further analyzed. A total of 584 enzymes were linked to protein folds using HMM superfamily prediction (⑤), 581 of which were painted with ancestry values (⑥). Overall, 1,255 enzymes were linked to protein folds and were colored in metabolic MANET (⑦).

Analysis and sorting of data in metabolic MANET

Figure S3 describes individual steps in the analysis and sorting of data. A total of 4,363 enzyme entries belonging to 137 metabolic pathways were registered in the KEGG pathway database file downloaded on December 2004. Out of these, 2,015 enzymes had subnetwork information. We assumed that these enzymes represent adequately the 132 metabolic subnetworks described in KEGG. Among these, 758 had structural PDB entries associated with them that could be used to assign SCOP folds architectures to metabolic nodes. We used a join operation to link 687 enzymes successfully to protein folds and 674 of them to ancestry values. The remaining 1,257 enzymes with no associated PDB entries and the 71 enzymes that could not be assigned to folds were subjected to HMM fold superfamily prediction (see Fig. S2A). As a result, 584 enzymes could be linked to folds, 581 of which were assigned ancestry values. A total of 1,255 enzymes were finally linked to protein folds and were painted in subnetworks. This represents 63% of all nodes in the metabolic network.

Contact information

THE MANET PROJECT, Atelier of Plant Bioinformatics, Department of Crop Sciences, University of Illinois, 1101 W Peabody Drive, Urbana, IL 61801, USA. Fax: 217-333-8046. E-mail: evolutionary-manet@uiuc.edu.

Table 1. Painting efficiency in metabolic MANET

KEGG pathway		N_{KEGG}	N_{MANET}	%
map00010	Glycolysis-gluconeogenesis	38	35	92.10
map00020	Citrate cycle (TCA cycle)	23	20	86.96
map00030	Pentose phosphate pathway	33	26	78.79
map00031	Inositol metabolism	5	5	100
map00040	Pentose-glucuronate interconversions	50	32	64
map00051	Fructose and mannose metabolism	58	37	63.79
map00052	Galactose metabolism	36	28	77.78
map00053	Ascorbate and aldarate metabolism	25	13	52
map00061	Fatty acid biosynthesis (path 1)	14	13	92.86
map00062	Fatty acid biosynthesis (path 2)	7	5	71.43
map00071	Fatty acid metabolism	28	20	71.43
map00072	Synthesis-degradation of ketone bodies	6	5	83.33
map00100	Biosynthesis of steroids	31	26	83.87
map00120	Bile acid biosynthesis	23	14	60.87
map00130	Ubiquinone biosynthesis	7	6	85.71
map00140	C21-Steroid hormone metabolism	16	11	68.75
map00150	Androgen and estrogen metabolism	23	14	60.87
map00190	Oxidative phosphorylation	11	9	81.82
map00193	ATP synthesis	1	1	100
map00195	Photosynthesis	3	3	100
map00220	Urea cycle-metabolism of amino groups	34	32	94.12
map00230	Purine metabolism	96	79	82.29
map00240	Pyrimidine metabolism	61	48	78.69
map00251	Glutamate metabolism	35	32	91.43
map00252	Alanine and aspartate metabolism	38	30	78.95
map00253	Tetracycline biosynthesis	3	1	33.33
map00260	Gly, Ser and Thr metabolism	55	47	85.45
map00271	Methionine metabolism	23	20	86.96
map00272	Cysteine metabolism	21	14	66.67
map00280	Val, Leu and Ileu degradation	31	24	77.42
map00290	Val, Leu and Ileu biosynthesis	15	13	86.67
map00300	Lysine biosynthesis	29	23	79.31
map00310	Lysine degradation	45	24	53.33
map00311	Penicillins-cephalosporins biosynthesis	8	6	75
map00330	Arginine and proline metabolism	67	46	68.66
map00340	Histidine metabolism	34	26	76.47
map00350	Tyrosine metabolism	62	37	59.68
map00360	Phenylalanine metabolism	38	24	63.16
map00361	γ-Hexachlorocyclohexane degradation	9	8	88.89
map00362	Benzoate degradation via hydroxylation	37	23	62.16
map00380	Tryptophan metabolism	52	33	63.46
map00400	Phe, Tyr and Trp biosynthesis	31	28	90.32
map00401	Novobiocin biosynthesis	6	6	100
map00410	beta-Alanine metabolism	32	19	59.37
map00430	Taurine and hypotaurine metabolism	14	7	50
map00440	Aminophosphonate metabolism	8	6	75
map00450	Selenoamino acid metabolism	21	18	85.71
map00460	Cyanoamino acid metabolism	18	10	55.56
map00471	D-Glutamine - D-glutamate metabolism	12	6	50
map00472	D-Arginine and D-ornithine metabolism	8	2	25
map00473	D-Alanine metabolism	6	4	66.67
map00480	Glutathione metabolism	27	13	48.15
map00500	Starch and sucrose metabolism	73	58	79.45
map00510	N-Glycan biosynthesis	25	17	68
map00511	N-Glycan degradation	8	8	100
map00512	O-Glycan biosynthesis	7	3	42.86
map00513	High-mannose N-glycan biosynthesis	2	2	100
map00520	Nucleotide sugars metabolism	29	13	44.83
map00521	Streptomycin biosynthesis	14	10	71.43
map00522	Biosynthesis of macrolides	2	1	50
map00523	Polyketide sugar unit biosynthesis	5	4	80
map00530	Aminosugars metabolism	36	28	77.78
map00531	Glycosaminoglycan degradation	11	9	81.82
map00532	Chondroitin-heparan sulfate biosynthesis	15	9	60
map00533	Keratan sulfate biosynthesis	5	4	80
map00540	Lipopolysaccharide biosynthesis	7	7	100
map00550	Peptidoglycan biosynthesis	16	14	87.5
map00561	Glycerolipid metabolism	75	50	66.67
map00562	Inositol phosphate metabolism	29	19	65.52
map00580	Phospholipid degradation	12	8	66.67
map00590	Prostaglandin-leukotriene metabolism	18	16	88.889
map00600	Glycosphingolipid metabolism	24	19	79.17
map00601	Blood glycolipid biosynthesis-lact.	8	5	62.5
map00602	Blood glycolipid biosynthesis-neolact.	11	7	63.64
map00603	Globoside metabolism	10	8	80
map00604	Ganglioside biosynthesis	6	4	66.67
map00620	Pyruvate metabolism	65	47	72.31
map00621	Biphenyl degradation	8	4	50
map00622	Toluene and xylene degradation	17	10	58.82
map00623	2,4-Dichlorobenzoate degradation	22	6	27.27
map00625	Tetrachloroethene degradation	3	2	66.67
map00626	Nitrobenzene degradation	11	5	45.45
map00627	1,4-Dichlorobenzene degradation	13	10	76.92
map00628	Fluorene degradation	7	5	71.43
map00629	Carbazole degradation	9	5	55.56
map00630	Glyoxylate-dicarboxylate metabolism	58	37	63.79
map00631	1,2-Dichloroethane degradation	4	4	100
map00632	Benzoate degradation via CoA ligation	27	18	66.67
map00640	Propanoate metabolism	43	29	67.44
map00641	3-Chloroacrylic acid degradation	2	2	100
map00642	Ethylbenzene degradation	3	2	66.67
map00643	Styrene degradation	15	10	66.67
map00650	Butanoate metabolism	48	33	68.75
map00660	C5-Branched dibasic acid metabolism	21	5	23.81
map00670	One carbon pool by folate	24	20	83.33
map00680	Methane metabolism	25	20	80
map00710	Carbon fixation	23	22	95.65
map00720	Reductive carboxylate cycle	13	13	100
map00730	Thiamine metabolism	13	8	61.54
map00740	Riboflavin metabolism	12	10	83.33
map00750	Vitamin B6 metabolism	20	6	30
map00760	Nicotinate and nicotinamide metabolism	28	19	67.86
map00770	Pantothenate and CoA biosynthesis	26	18	69.23
map00780	Biotin metabolism	11	10	90.91
map00790	Folate biosynthesis	24	22	91.67
map00791	Atrazine degradation	7	4	57.14
map00830	Retinol metabolism	10	1	10
map00860	Porphyrin and chlorophyll metabolism	57	44	77.19
map00900	Terpenoid biosynthesis	15	9	60
map00901	Indole and ipecac alkaloid biosynthesis	22	2	9.09
map00902	Monoterpenoid biosynthesis	16	1	6.25
map00903	Limonene and pinene degradation	10	3	30
map00904	Diterpenoid biosynthesis	18	8	44.44
map00910	Nitrogen metabolism	55	44	80
map00920	Sulfur metabolism	29	19	65.52
map00930	Caprolactam degradation	9	4	44.44
map00940	Stilbene, coumarine, lignin biosynthesis	19	9	47.37
map00941	Flavonoid biosynthesis	20	9	45
map00950	Alkaloid biosynthesis I	37	7	18.92
map00960	Alkaloid biosynthesis II	10	6	60
map00970	Aminoacyl-tRNA biosynthesis	21	21	100
map01051	Biosynthesis of ansamycins	1	1	100
map01053	Biosynthesis of siderophore group	4	4	100
map01055	Biosynthesis of vancomycin antibiotics	1	1	100
map02040	Flagellar assembly	1	1	100
map03020	RNA polymerase	1	1	100
map03030	DNA polymerase	1	1	100
map03050	Proteasome	1	1	100
map03060	Protein export	2	2	100
map03070	Type III secretion system	1	1	100
map03090	Type II secretion system	2	2	100
map04070	Phosphatidylinositol signaling system	23	18	78.26
map04120	Ubiquitin mediated proteolysis	1	1	100

N, number of nodes present in each subnetwork and painted by MANET
